# Genetic diversity of *Bertholletia excelsa*, an Amazonian species of wide distribution

**DOI:** 10.1186/1753-6561-5-S7-P5

**Published:** 2011-09-13

**Authors:** Patrícia Sujii, Karina Martins, Lucia Wadt, Vânia Azevedo, Vera Solferini

**Affiliations:** 1 Laboratório de Diversidade Genética, Universidade Estadual de Campinas, Campinas, Brazil; 2 Universidade Federal de São Carlos, Brazil; 3 Embrapa Acre, Brazil; 4 Laboratório de Genética Vegetal, Embrapa Recursos Genéticos e Biotecnologia, Brazilia, Brazil

## Background

Amazonian upland forests are expansive and can comprise large continuous tracts. There have been several studies on the population genetic structure of species in this kind of forest, but there are few studies that aim to understand genetic structure throughout the Amazon [1]. The Brazil-nut tree, *Bertholletia excelsa*, is a monotypic genus, endemic to upland forests and distributed along almost the entire expanse of the Amazon [2-5].

Genetic diversity distribution through Amazon forest is an understudied issue, specially in plants. Quantifying and understanding population genetic structure, associated to gene flow and mating system studies are recognized as important tools for the development of strategies for conservation and management. Such information can also be helpful in identifying effects of habitat fragmentation [6,7].

This study aimed to evaluate genetic structure of *B. excelsa* populations in the Amazon and to verify if the structuring is influenced by distance between them.

## Methods

Seven polymorphic microsatellite markers were developed from a dinucleotide-enriched genomic library. Material from 379 individuals was collected from nine Amazonian subpopulations distributed in five Brazilian states (AC: Acre, AM: Amazonas, AP: Amapá, PA: Pará, RR: Roraima). All seven microsatellite markers developed for the species were used with four others previously published markers [8], for genotyping.

Analyses within and among populations were performed to evaluate genetic diversity and population structure. All nine populations were characterized with 11 microsatellite loci for number of alleles per locus, allele frequency and observed and expected heterozygosity under Hardy-Weinberg expectation. Wright fixation indices were also estimated. Genetic distance estimates were correlated with different potential factors to find possible causes of genetic structure.

## Results and conclusions

A few alleles were found in each subpopulation and considerable variation was observed in alleles found in each subpopulation and in their allele frequencies, especially when compared with very distant subpopulations. Heterozygote excess was observed in five subpopulations while in the other four subpopulations, estimates of *F_IS_*_,_ non-significantly different from zero, were found. The observed high heterozygote proportions in most subpopulations and absence of inbreeding support the existence of self-incompatibility mechanisms and selection in favor of heterozygotes. Maintenance of genetic variability is favored by the alogamous breeding system, which is an important feature to be considered in conservation and management strategies, in order to avoid fertility deficits.

Fine-scale structure, when present, was small. Estimates of inter population genetic structure varied from low (*q*= 0,02) to high (*q*= 0,244). *B. excelsa* genetic structure can be analyzed on three different scales: (*i*) within population; (*ii*) among moderately distant populations (<500km); and (*iii*) among very distant subpopulations. At all scales, significant correlations were found between genetic structure and geographic distance between pairs of individuals or populations. This may indicate that distance is an important factor in this population’s genetic structure, but probably there are other factors acting in conjunction, especially on a large geographical scale.
